# Supplementary material for: Modulation of Campylobacter jejuni Motility, Adhesion to Polystyrene Surfaces, and Invasion of INT407 Cells by Quorum-Sensing Inhibition
Source: Microorganisms. 2020 Jan 11;8(1):104. doi: 10.3390/microorganisms8010104 (PMC7022965; doi:10.3390/microorganisms8010104)
Supplement: Supplementary file 1 [file microorganisms-08-00104-s001.pdf]

Supplementary Information

Supplementary Table S1. Details of the treatments used in this study, with their corresponding antimicrobial activities.

Preparation	Code	Plant/ substance	Source	MIC (mg/L)	
				**1.0×**	**0.25×**
Essential oils	EO1	*Juniperus communis*	Herbana d.o.o., Slovenia	250	62.5
	EO2	*Origanum* sp.	Lek Veterina d.o.o., Slovenia	62.5	15.62
	EO3	*Syzygium aromaticum*	Lek Veterina d.o.o., Slovenia	250	62.5
	EO4	*Rosmarinus officinalis*	Lek Veterina d.o.o., Slovenia	125	31.25
	EO5	*Lavandula hybrida*	M. Jeršek s.p., Slovenia	250	62.5
	EO6	*Thymus vulgaris*	Lek Veterina d.o.o., Slovenia	62.5	15.62
Ethanolic extracts	E1 (pre-distillation)	*Origanum* sp.	This study	250	62.5
	E2 (post-distillation)	*Origanum* sp.	This study	250	62.5
	E3 (leaves)	*Origanum* sp.	This study	500	125
	E4 (flowers)	*Origanum* sp.	This study	500	125
	E5 (pre-distillation)	*Urtica dioica*	This study	1000	250
	E6 (post-distillation)	*Urtica dioica*	This study	1000	250
	E7	*Satureja montana*	This study	250	62.5
	E8	*Sedum rosea*	Alperth et al. 2019	500	125
	E9 (pre-distillation)	*Achillea millefolium*	This study	1000	250
	E10 (post-distillation)	*Achillea millefolium*	This study	1000	250
	E11	*Rosmarinus officinalis*	This study	500	125
Pure compounds	P1	Carvacrol	Sigma	31.25	7.81
	P2	Rosmarinic acid	Sigma	250	62.5
	P3	*y*-Terpinene	Sigma	125	31.25

Supplementary Table S2. *C. jejuni* 11168 motility (colony diameter in mm), adhesion on polystyrene surfaces (log_{10} CFU/well) and invasion of and adhesion to INT407 cells (log_{10} CFU/mL) in un-treated culture (Control) and in treated cultures. Results are presented as mean ± standard deviation (SD).

Treatment code	Motility		Adhesion on polystyrene surfaces		Invasion of INT407		Adhesion of INT407	
	Diameter in mm	SD	log_{10} CFU/well	SD	log_{10} CFU/mL	SD	log_{10} CFU/mL	SD
Control	78	1	7.19	0.05	4.75	0.06	5.79	0.20
EO1	74	2	5.30	0.05	4.12	0.23	5.54	0.23
EO2	66	9	7.23	0.10	4.13	0.25	5.36	0.20
EO3	66	3	6.51	0.13	4.14	0.11	5.73	0.26
EO4	64	8	6.14	0.26	4.10	0.17	5.51	0.12
EO5	67	5	5.75	0.07	3.83	0.25	5.54	0.10
EO6	73	6	6.83	0.39	3.82	0.32	5.54	0.11
E1	73	3	7.12	0.04	3.96	0.46	5.50	0.26
E2	69	5	7.05	0.04	3.90	0.38	5.52	0.25
E3	69	8	6.85	0.18	3.93	0.33	5.42	0.38
E4	70	1	6.99	0.07	3.96	0.47	5.11	0.17
E5	71	6	7.13	0.25	4.18	0.45	5.40	0.06
E6	72	2	7.19	0.11	3.88	0.46	5.35	0.28
E7	72	5	7.11	0.13	4.04	0.14	5.21	0.14
E8	51	11	5.77	0.22	4.01	0.35	5.55	0.25
E9	76	2	6.82	0.38	3.76	0.37	5.38	0.07
E10	74	7	6.76	0.32	4.01	0.31	5.49	0.11
E11	64	13	7.26	0.10	3.76	0.26	5.40	0.13
P1	74	8	6.94	0.25	3.73	0.34	5.35	0.30
P2	71	3	7.18	0.02	4.18	0.23	5.34	0.24
P3	68	3	6.16	0.06	4.07	0.21	5.16	0.23

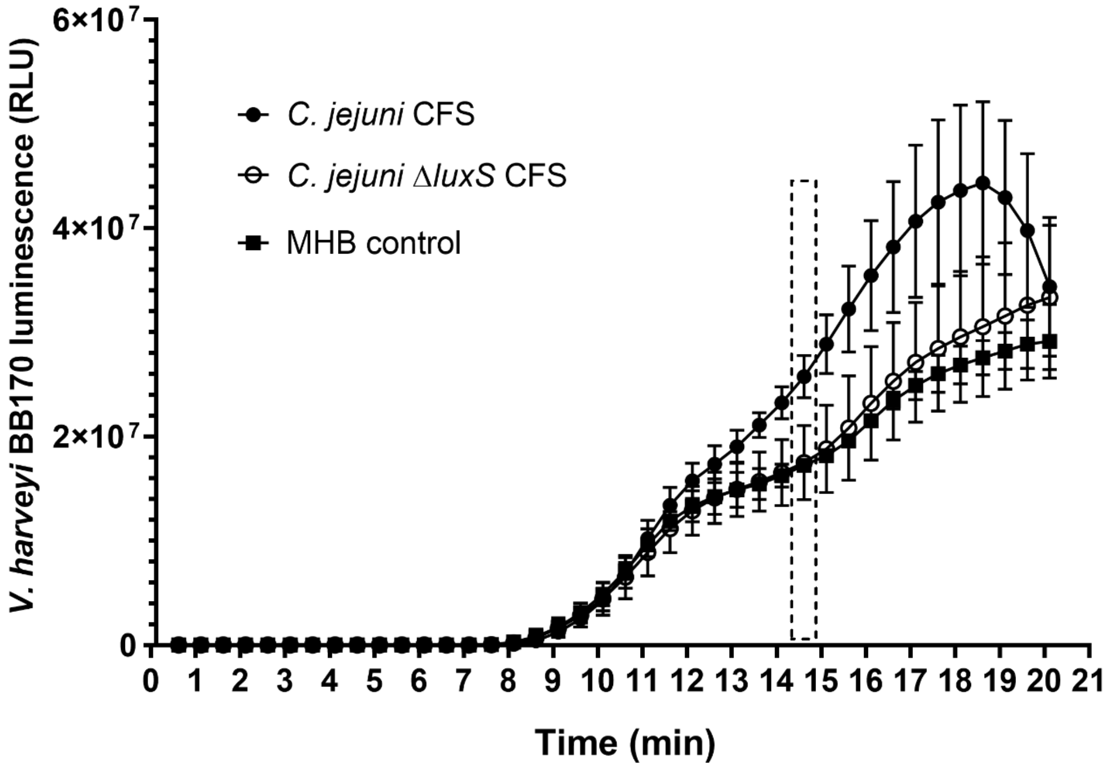

Supplementary Figure S1. *V. harveyi* BB170 luminescence production after addition of *C. jejuni* 11168 and *C. jejuni* 11168Δ*luxS* cell-free supernatants, and the Mueller Hinton broth control (MHB). Data are means ±standard deviation of kinetic measurements every 30 min. Dashed box, time point used for further quorum-sensing inhibition measurements (14.5 h); RLU, relative luminescence units.

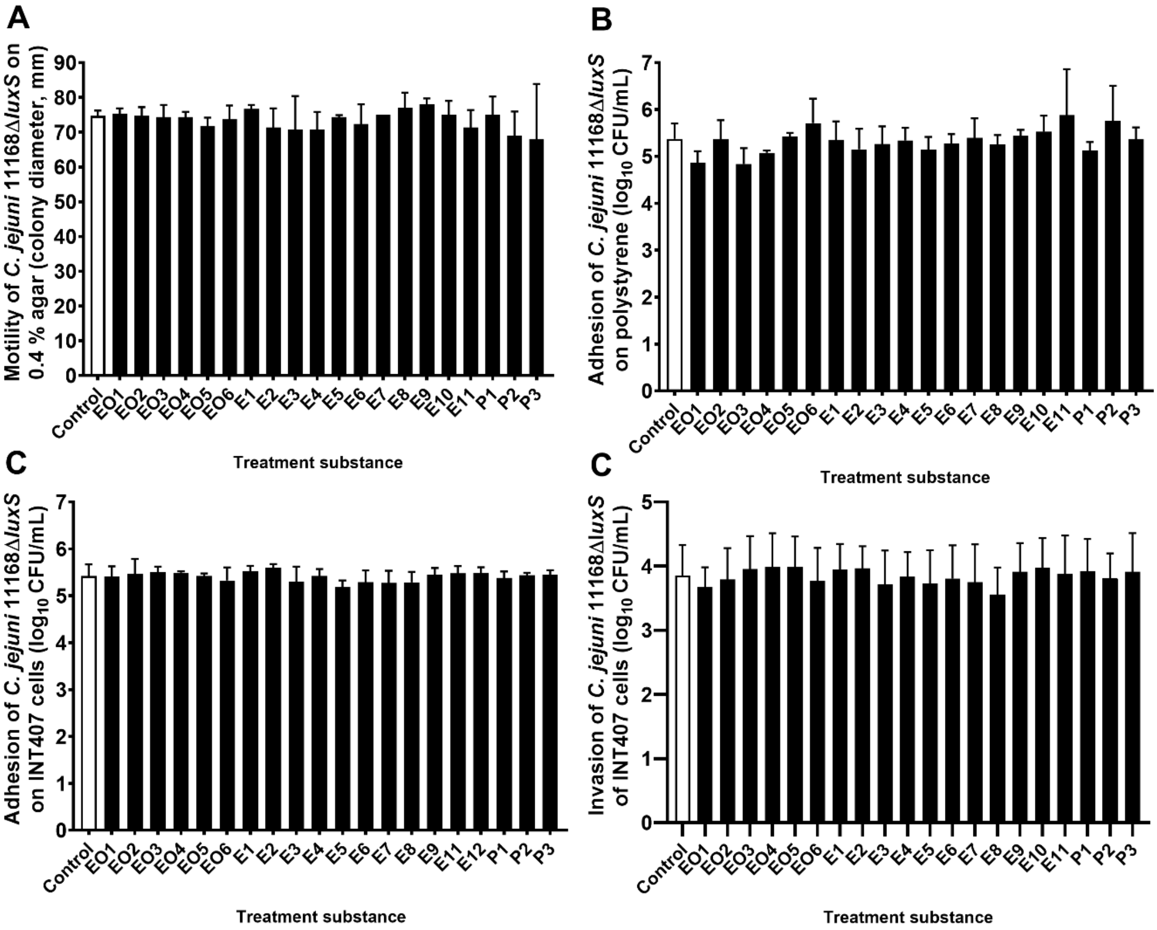

Supplementary Figure S2. *C. jejuni* 11168ΔΔ*luxS* (A) motility on 0.4 % agar (colony diameter, mm), (B) adhesion on polystyrene (log10 CFU/well), (C) adhesion and (D) invasion of INT407 cells (CFU/mL), without (Control, empty bar) and with treatments with essential oils (EO1-6), ethanolic extracts (E1-11), and pure compounds (P1-3) (full bars). Data are means ±standard deviations.
